# "Going Smoke Free" – BMA booklet review

**DOI:** 10.1186/1617-9625-3-2-3

**Published:** 2006-08-15

**Authors:** Samuel Hawkins

**Affiliations:** 1 University of Louisville, Kentucky, USA

## 

*Going smoke-free*[1] from the Tobacco Advisory Group of the Royal College of Physicians presents a well thought out and documented approach to the "medical case" for establishing smoke-free environments at home, work, and in public areas. It addresses the medical evidence and present a convincing argument in favor of smoke-free policy. The authors conclude from their information that enacting "legislation to make all workplaces and public spaces smoke-free" should be pursued as quickly as possible citing many medical benefits as well as economic spin-offs and public support. However, it is not medical concerns that directly stimulate passage of legislation but rather political concerns, which are informed by medical, as well as practical, social, economic and ethical issues. Therefore *Going Smoke Free *devotes the first four chapters specifically to the medical case for legislation supporting smoke-free environments. These address a comprehensive array of medical conditions that appear to be related in one manner or another to environmental tobacco smoke (ETS). The remaining chapters provide interesting information and compelling arguments in favor of smoke-free policies that support the political case for legislation and address many of the tobacco industry's tactics to negate the anti-smoking criticisms they have endured in the recent past. Going smoke free is a comprehensive primer that balances critical analyses of the evidence in a format that is concise and accessible. The discussions are well cited, and the lists of references alone will be resources to advocates of smoke-free policy.

This publication from the Royal College of Physicians brings together substantial epidemiological research from several countries providing the evidence that overwhelmingly supports the institution of smoke-free policies. While many studies have shown that direct smoking induces a wide array of health problems, few other all-encompassing works are available particularly related to second hand smoke exposure. Environmental tobacco smoke exposure has been studied sufficiently over the last few years for links between ETS and a number of diseases to be evident. ETS increases by between 20 and 40 percent the risk of developing lung cancer, ischaemic heart disease, COPD, and stroke. *Going smoke-free *applies to these data a published statistical model to estimate the number of deaths from the above four diseases that can be attributed to ETS exposure, and they estimate that almost 500 deaths per year can be attributed solely to ETS exposure in the workplace.

Total smoking restrictions and smoke-free policies that are in place are shown to be effective at almost eliminating exposure to ETS in the workplace and public spaces. One study in New York City showed an average decrease in respiratory suspended particles of 93 percent in a variety of bars and restaurants following the city's total indoor smoking ban. Another study from Helena, Montana, suggests that six months into a smoking ban, a reduction in hospital admissions for myocardial infarction for residents who worked and lived inside the city had occurred, whereas there was no corresponding decrease in admissions from the group who lived outside of the city.

The British public appears appraised of the dangers of ETS exposure, with 8 of 10 people agreeing that breathing someone else's smoke increases their risk of lung cancer, bronchitis, and asthma. This likely contributes to the high public support for smoke-free legislation among the British public. A Philip Morris study from as early as 1989 suggested that 70 percent of adults in the UK believe the government should pass laws restricting smoking in public places. Today support lies above 80 percent, though it is less for smoking restrictions in pubs. The evidence from extant smoking bans in the US and Ireland indicate that their implementation can be met with widespread public support. In Ireland after implementation of smoking restrictions in pubs almost 50% of smokers support such a ban compared to very low levels prior to activation.

The case for smoke-free policies should be sufficient to spur passage of legislation. However, the debate is heavily influenced by the tobacco industry, which is keen to prevent passage of legislation. *Going smoke-free *devotes a very interesting chapter to the history of the tobacco industry's involvement in the debate over smoke-free policies, detailing the tactics used to dilute and divert attention from the strong case in favor of total smoking bans. The industry's tactics include enflaming merchants' and governments' fears of dire economic consequences, sponsoring groups to lobby for "smokers' rights", and sponsoring scientific groups to challenge the evidence for links between ETS and disease. *Going smoke-free *addresses each of these claims and rhetorical tricks of the tobacco industry. The discussion of the lobby for "smokers' rights" while not extensive provides some ethical balance in addressing the dilemma of these rights versus non-smokers' rights to clean air.

Ethical and civil-liberties arguments of the concept of smokers' rights are unpacked, and skillfully disputed. Two chapters are devoted to the economic impact of smoke-free policy, one of which is devoted to its effect on the hospitality industry (pubs and restaurants) and disputes the recurring claims that smoking bans have negative economic consequences. An excellent discussion is also included of how the scientific literature is appraised by the tobacco industry and its attempts to suggest that what has been done to date is not "sound science". Opponents of smoke-free legislation exploit this fact, posing irresponsible challenges to the scientific evidence, making sweeping claims that the evidence is not yet clear, and based on isolated studies, ETS is innocuous. It is true that biases affect claims made about particular studies, but those biases can both over- and underestimate the link between ETS and disease. Claims about the scientific evidence must be made by appraising the full body of research performed on a particular topic, something aided by the publishing of systemic overviews and meta-reviews. Overviews that critique the available literature in its entirety are a far more reliable indicator than the results of single studies. Indeed an excellent example of the latter are the is the consistent findings of relative risks for developing lung cancer in never-smokers exposed to ETS.

With the existing and pending comprehensive smoke-free legislation in the UK, the tobacco industry will continue to attempt to thwart and dilute the legislation. For those wishing to see the full implementation of smoke-free legislation in the UK, and for other nations considering the passage of smoke-free legislation, this book is an invaluable resource.

## Competing interests

The authors declare that they have no competing interests.
